# A new leafhopper genus of Empoascini (Hemiptera, Cicadellidae, Typhlocybinae) from China

**DOI:** 10.3897/zookeys.908.48316

**Published:** 2020-01-30

**Authors:** Xiaofei Yu, Rui Shi, Maofa Yang

**Affiliations:** 1 College of Tobacco Science, Guizhou University, Guiyang, Guizhou, 550025, China; 2 Institute of Entomology, Guizhou University, Guiyang, Guizhou, 550025, China; 3 Guizhou Key laboratory of Tobacco Quality Research, Guiyang, Guizhou, 550025,China

**Keywords:** Auchenorrhyncha, Empoascini, taxonomy, new genus, China

## Abstract

A new leafhopper genus, *Fanjinga***gen. nov.**, with type species *F. digitata* Yu & Yang, **sp. nov.**, is described from the Guizhou Province of Southwest China. Habitus photos and illustrations of male genitalia of the new species are provided.

## Introduction

The *Alebroides* group of Empoascini leafhoppers includes 25 genera and 156 species, widely distributed in the Palaearctic Region and Old World tropics (Xu et al. 2016). Of these taxa, 16 genera and 60 species are known from China (Xu et al. 2016, 2017, Yu and Yang 2013, 2014a, b). A key to the group, characterized by the branched CuA vein in the hind wing (Fig. 13), was given by Xu et al. (2017). This paper adds a new genus with one new species, based on material from Guizhou (Southwest China).

## Materials and methods

Body measurements are from apex of vertex to tip of forewing. Habitus photos were taken using a VHX-5000 digital microscope; illustrations of male genitalia were inked in Adobe Illustrator CS6, then digitized (300dpi). For morphological terminology we mostly follow Zhang (1990), but the wing venation follows Dworakowska (1993), the legs follow Dietrich (2005) and chaetotaxy of the subgenital plate follows Southern (1982). The new specimens examined are deposited in Institute of Entomology, Guizhou University, Guiyang, China (GUGC) and School of Karst Science, Guizhou Normal University, Guiyang, China.

## Taxonomy

### Fanjinga

Taxon classificationAnimaliaHemipteraCicadellidae

Yu & Yang gen. nov.

04EA95A4-B242-55E4-AD37-69DA953F1F0F

http://zoobank.org/F1EB5835-F39D-4C1A-862C-3F55C8618661

#### Type species.

*Fanjinga digitata* Yu & Yang, sp. nov., here designated.

#### Diagnosis.

Within the *Alebroides* group of Empoascini, the new genus runs to *Nulliata* Lu, Xu & Qin in the key to genera by Xu et al. (2017), based on the following characters (in order of couplets): MP’ vein arising from r cell, coronal suture not extending onto face, anal tube appendage absent, crown face transition without a dark medial patch and male pygofer not emarginated dorsally, aedeagus with preatrium long. The new genus differs from *Nulliata* in having the abdominal apodemes long and from *Nulliata* and other genera in having a finger-like process bearing micro-setae on the lower posterior margin of the male pygofer.

#### Description.

**Figures 1–11.**
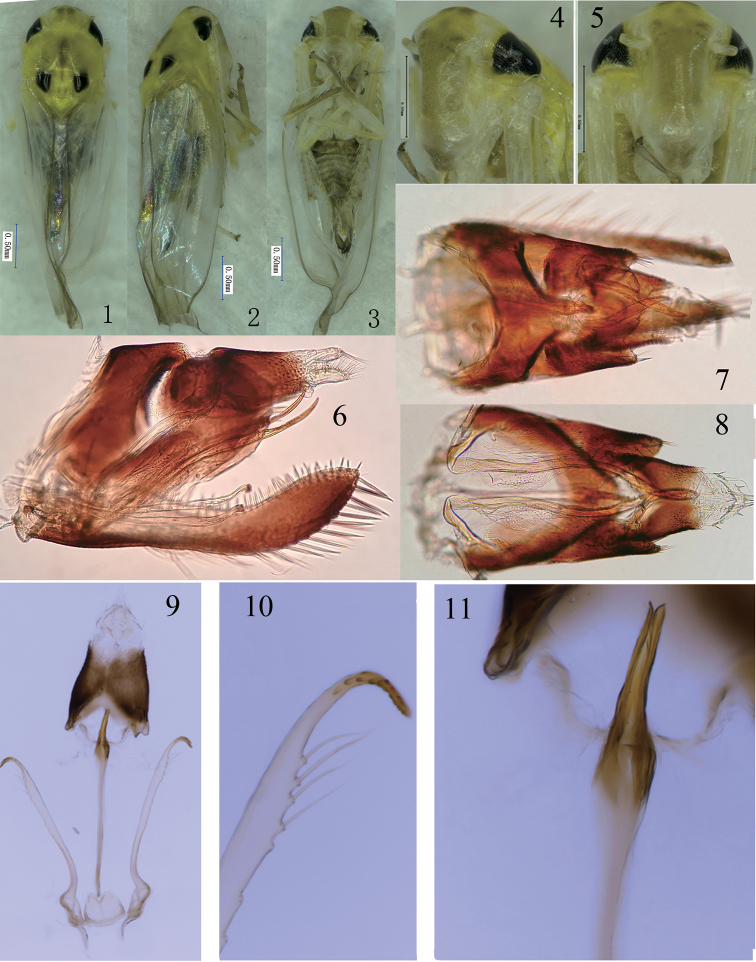
*Fanjinga digitata*, sp. nov. **1** male adult, dorsal view **2** male adult, lateral view **3** male adult, ventral view **4** head and thorax, ventrolateral view **5** head and thorax, ventral view 6 male genitalia, lateral view **7** male genitalia, dorsal view **8** male genitalia, ventral view **9** anal tube, connective and pygofer, ventral view **10** paramere, terminal part **11** aedeagus, terminal part. Scale bars: 0.5mm.

**Figures 12–23.**
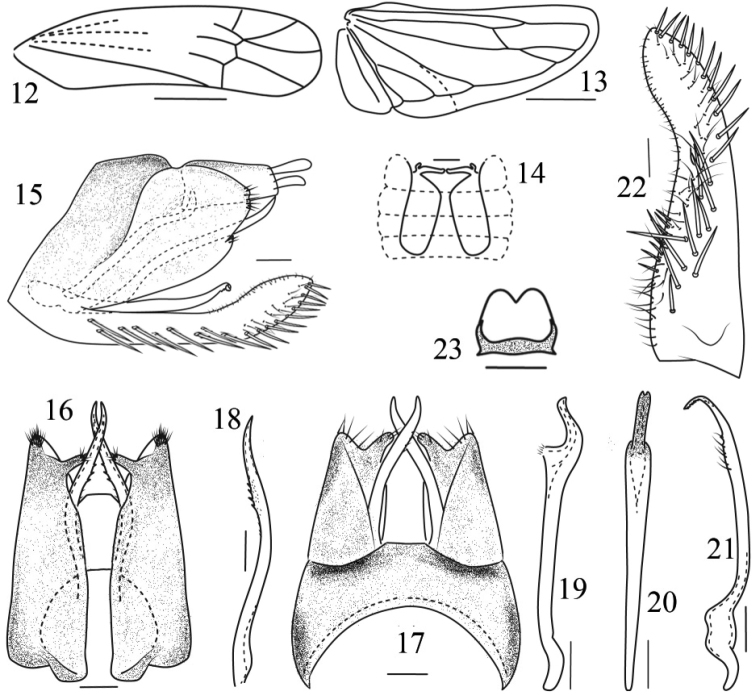
*Fanjinga digitata*, sp. nov. **12** forewing **13** hindwing **14** abdominal apodemes **15** male genitalia, lateral view **16** pygofer, ventral view **17** pygofer, dorsal view **18** ventral pygofer appendage **19** aedeagus, lateral view **20** aedeagus, ventral view **21** paramere, lateral view **22** subgenital plate, lateral view **23** connective, dorsal view. Scale bars: 0.1mm.

Relatively robust (Figs 1–3). Head slightly narrower than pronotum; vertex short, slightly longer medially than next to eyes, coronal suture reaching to anterior margin of vertex. Ocelli present. Face broad, distinctly broadened at lower part, convex in profile (Figs 3–5). Pronotum nearly twice as long as vertex (Fig. 1). Forewing with apical cells occupying almost 1/3 of total length, veins RP and MP’ stalked at base, arising from r cell, and MP’’+CuA’ from m cell, r cell longer than m cell, but narrower than m cell, third apical cell triangular (Fig. 12). Hindwing with CuA branched, termination of branch distad of coalescence of CuA with MP’’ (Fig. 13). Front femur with AM1 enlarged, with one antero-basal macroseta and 8 small setae near tip of femur. Hind femur with macrosetae 2+1+1, hind tibia row AV with 10 macrosetae near apex.

Male basal abdominal sternal apodemes well developed (Fig. 14). Male pygofer elongate, dorsolateral fracture distinct, dorsal bridge moderately long, with several rigid microsetae at posterior margin; ventral appendage well developed, extended beyond posterior margin; lower posterior margin with a finger-like process (Figs 6–8, 15–18). Subgenital plate extended slightly beyond pygofer, A-group setae present, B-group setae following A-group setae to tip of dorsal margin, C-group setae arranged in two rows in basal half, then merged into single row to apex; D-group setae thick (Fig. 22). Paramere very thin, curved apically, apex with teeth and sensory pits (Fig. 10, 21). Aedeagus slender, preatrium very long, dorsal apodeme absent (Figs 9, 11, 19–20). Anal tube appendage absent (Fig. 9). Connective almost square shaped, apical margin medially concave (Fig. 23).

#### Etymology.

The genus name is an arbitrary combination of letters.

#### Distribution.

China (Guizhou).

### Fanjinga digitata

Taxon classificationAnimaliaHemipteraCicadellidae

Yu & Yang sp. nov.

FE13424B-E8A8-5677-B624-2DA047EAD319

http://zoobank.org/F427C9A4-AEFC-472A-AFAB-B158C408A80E

#### Description.

Size. Male 3.94–4.00mm.

Whole body yellowish, anterior margin of vertex marked with two brownish patches (Figs 1–3). Pronotum with a longitudinal streak centrally (Figs 1, 2). Scutellum with basolateral triangles black (Figs 1, 2). Abdomen yellowish (Fig. 3). Legs yellowish, except tibia; tarsus and claws of front legs brownish.

Basal sternal abdominal apodemes extending to end of segment VI (Fig. 14). Male pygofer with microsetae at posterior margin, pygofer ventral appendages with teeth on dorsal side, extending beyond posterior margin; lower posterior margin with a finger-like process bearing several microsetae; dorsal bridge occupying more than 1/3 length of pygofer in dorsal view (Figs 6–8, 15–18). Subgenital plate with basal 2/3 curved dorsad in lateral view, with 7 A-group setae, ca. 30 B-group setae, 23 C-group setae and ca. 49 D-group setae (Fig. 22). Paramere with few microsetae and sensory pits subapically (Figs 10, 21). Aedeagus shaft curved dorsad, gonopore apical; preatrium very long, almost 3 times length of shaft; dorsal apodeme absent (Figs 19, 20). Connective notched medially at apex, slightly broader at base (Fig. 23).

#### Holotype.

♂, China, Guizhou, Fanjing Mountain, 21-August-2017, coll. Yarong Gao (GUGC). Paratypes: 3♂, same collecting data as holotype (1♂ GUGC, 2♂ School of Karst Science, Guizhou Normal University).

#### Etymology.

The new species is named from the Latin “digitalis”, meaning finger, for the finger-like process of the male pygofer.

#### Host plants.

*Euonymus* (Celastraceae).

#### Distribution.

China (Guizhou).

## Supplementary Material

XML Treatment for Fanjinga

XML Treatment for Fanjinga digitata
